# Impact of 1-week preoperative auto-CPAP treatment on postoperative outcomes in patients undergoing heart valve replacement surgery: a prospective randomized controlled trial

**DOI:** 10.3389/fneur.2023.1152168

**Published:** 2023-06-05

**Authors:** Mei Su, Wei Lin, Qi Xu, Buqing Ni, Xilong Zhang, Shijiang Zhang, Ning Ding

**Affiliations:** ^1^Department of Respiratory and Critical Care Medicine, The First Affiliated Hospital of Nanjing Medical University, Nanjing, China; ^2^Department of Geriatrics, The First Affiliated Hospital of Nanjing Medical University, Nanjing, China; ^3^Department of Oncology, The First Affiliated Hospital of Nanjing Medical University, Nanjing, China; ^4^Department of Cardiovascular Surgery, The First Affiliated Hospital of Nanjing Medical University, Nanjing, China

**Keywords:** obstructive sleep apnea, continuous positive airway pressure (CPAP), cardiac valve replacement, postoperative complication, hospital stay

## Abstract

**Background:**

Whether preoperative continuous positive airway pressure (CPAP) treatment improves postoperative outcomes in patients undergoing cardiac valve replacement (CVR) remains unknown.

**Hypothesis:**

This study was to evaluate the effects of 1-week perioperative auto-continuous positive airway pressure (CPAP) treatment on postoperative heart and pulmonary outcomes in patients with obstructive sleep apnea (OSA) and valvular heart disease.

**Methods:**

Thirty-two patients with OSA and valvular heart disease were randomly assigned to 1-week CPAP (*n* = 15) group and non-CPAP treatments (*n* = 17) group. After the treatment, all patients underwent CVR surgery. The length of ICU and hospital stays, postoperative cardiac and respiratory complications were assessed and compared between the 2 groups.

**Results:**

The results showed there was no significant difference in the baseline characteristics between the CPAP and non-CPAP treatment groups. The length of postoperative ICU and hospital stays, as well as the duration of mechanical ventilation were significantly reduced in the CPAP treatment group compared to the non-CPAP treatment group; however, there were no significant differences in cardiac complications (postoperative arrhythmias, pacemaker use, first dose of dopamine in the ICU, and first dose of dobutamine in the ICU), and respiratory complications (reintubation and pneumonia).

**Conclusion:**

We concluded that in patients underwent CVR, preoperative use of auto-CPAP for OSA significantly decreased the duration of mechanical ventilation, and postoperative stays in the ICU and hospital.

**Clinical Trial Registration**: https://ClinicalTrials.gov, identifier NCT03398733.

## 1. Introduction

Obstructive sleep apnea (OSA) is one of the most common sleep disorders in adults, with reported prevalence rates between 16 and 84% (1, 2). OSA is associated with increased all-cause and cardiovascular mortality risks, as well as the incidence of hypertension (3), coronary heart disease, heart failure and postoperative cardiovascular events (sudden cardiac death, atrial fibrillation, congestive heart failure, and myocardial injury) (4–6). In our previous study we reported that OSA is common in patients undergoing cardiac valve replacement (CVR). Moreover, preoperative OSA is associated with an increased mechanical ventilation duration and a longer intensive care unit (ICU) stay, as well as a higher rate of postoperative pacemaker use (7).

Continuous positive airway pressure (CPAP) is the mainstay treatment for patients with OSA. A previous study showed that the postoperative complications are increased in patients with untreated OSA who undergo general and vascular surgery (8). Although it is well-established that perioperative CPAP significantly reduces the postoperative apnea-hypopnea index (AHI) and pulmonary complications, improves the oxygen saturation, and shortens the length of hospital stay following cardiac and non-cardiac surgery (9–12); however, it is not completely clear whether perioperative CPAP improves the postoperative outcomes in patients undergoing CVR surgery.

The objective of this study was to determine if perioperative CPAP treatment could improve postoperative adverse events, including decreasing the lengths of stay (hospital, preoperative, postoperative, and ICU), cardiac complications (postoperative arrhythmias, pacemaker use, first dose of dopamine in the ICU, and first dose of dobutamine in the ICU), and respiratory complications (reintubation, pneumonia, and duration of mechanical ventilation).

## 2. Methods

### 2.1. Subjects and study design

Between January 2018 and December 2019, a total of 223 patients with rheumatic valvular heart disease (18–75 years of age) undergoing CVR were screened for OSA using ApneaLink^™^ Air (ResMed Corp., Australia) in the first 3 days of hospitalization. Patients with OSA who agreed to participate in our study underwent an overnight polysomnography (PSG) the next day. The patients with an AHI ≥ 5/h were randomly assigned to the CPAP (*n* = 17) and non-CPAP treatment groups (*n* = 17). Patients in the CPAP treatment group received CPAP + basic treatment for 7 d (15 patients completed the 7-d treatment). Patients in the non-CPAP treatment group received basic treatment for 7 d. All patients underwent CVR surgery 1 week after completing treatment. The patients were admitted to the ICU and received mechanical ventilation postoperatively. The postoperative PSG was performed 1–3 d prior to hospital discharge.

**Figure 1:**
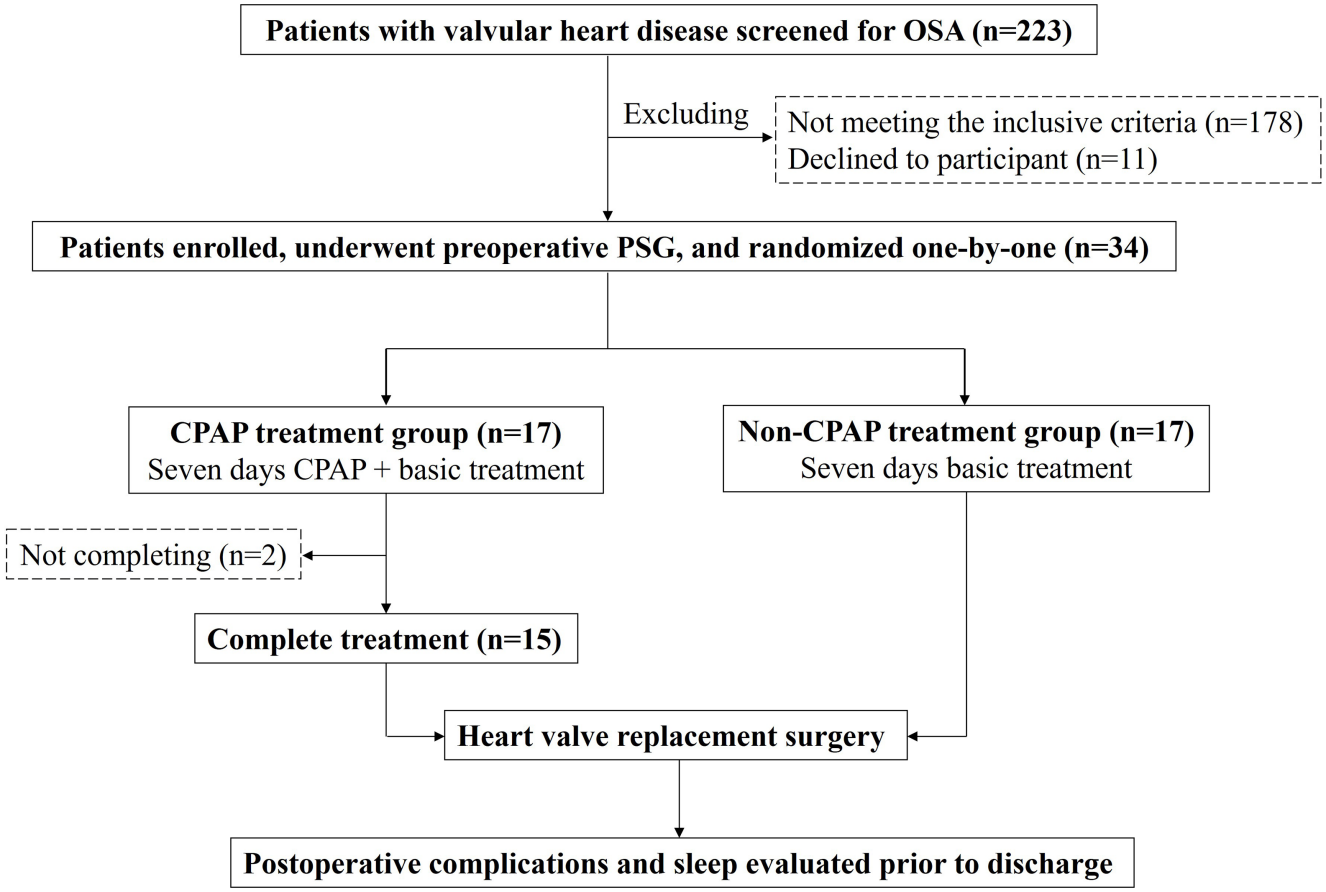
Flowchart of patients enrollment.

The inclusion criteria were as follows: (1) 18–75 years of age, (2) diagnosed with rheumatic valvular heart disease, (3) diagnosed with OSA (AHI > = 5/h), (4) received heart valve replacement surgery, and (5) signed informed consent. The exclusion criteria were as follows: (1) history of stroke or clinical signs of peripheral or central nervous system disorders, (2) previously known congenital heart disease, coronary heart disease, myocardial infarction, dilated cardiomyopathy, or hypertrophic cardiomyopathy, and (3) chronic obstructive pulmonary disease or a history of asthma. Figure 1 shows the study design flow chart.

#### 2.1.1. Randomized method

Random sequence numbers (1–34) were generated using Excel software by a statistician who did not otherwise take part in this study. According to the order of enrollment, the random sequence number was used one-by-one. Odd numbers were assigned to the CPAP treatment group and even numbers were assigned to the non-CPAP treatment group. The statistician and the data collector were blinded to interventions and the care providers were blinded to outcome parameters.

Preoperative basic treatment included oxygen and optimal medications. Oxygen therapy was administered when a patient had a pulse oxygen saturation (SpO_2_) < 90%. The optimal medication therapy included digoxin, diuretics, nitrates, an angiotensin-converting enzyme inhibitor/angiotensin receptor blocker, and a *β*-blocker.

The diagnosis of rheumatic valvular heart disease was based on the 2012 criteria for the diagnosis of rheumatic fever and rheumatic heart disease, as follows (13): a primary episode of rheumatic fever or current clinical rheumatic heart disease as featured and typical rheumatic valvular lesions were examined by Doppler echocardiography.

### 2.2. Baseline clinical evaluation

The preoperative physical examinations and heart function evaluations were performed by the same physician. Patient height, weight, and body mass index (BMI [weight in kg/height in m^2^]) were recorded. The New York Heart Association (NYHA) classification was determined. Atrial fibrillation was detected by 12-lead electrocardiography. Two-dimensional Doppler echocardiography was performed to assess left ventricular ejection fraction (LVEF). Valvular disease was assessed by echocardiography and confirmed by pathologic evaluation postoperatively. The 6-min walking test (6MWT) was performed within 3 d after hospital admission, as we previously reported (7, 14).

### 2.3. Polysomnography

The Epworth Sleepiness Scale (ESS) score was recorded before the sleep study to assess daytime sleepiness. The sleep study was an unattended overnight PSG (Alice 6 LDx System; Respironics, Inc., Netherlands) pre-and postoperatively. We used the 2012 standards of the American Academy of Sleep Medicine (AASM) (15) to score sleep apnea (SA) types and associated events, as described previously (7): obstructive apnea, >90% decrease in airflow with continued paradoxical chest and abdominal excursion for ≥10 s; central apnea, >90% decrease in airflow as well as complete cessation of chest and abdominal excursion ≥10 s; and hypopnea-reduction of airflow, >50% baseline lasting ≥10 s and associated with ≥3% desaturation. The AHI was defined as the number of apneic and hypopnea events per h of sleep. An AHI of ≥5/h was considered diagnostic for SA. Sleep apnea in which greater than 50% of events were obstructive, was defined as OSA.

### 2.4. CPAP treatment

An auto-CPAP (S9 Auto Set; ResMed Corp., Australia) treatment regimen (auto-CPAP, 4–16 cm H_2_O) was offered to the patients in the CPAP treatment group. Recordings with a mean CPAP use >4 h/night were accepted for each patient (16). To prevent confounding, all patients used the same CPAP device, and underwent the same clinical evaluations, follow-up assessments, and education regarding OSA and proper CPAP use. All patients had formal mask fitting prior to initiating CPAP therapy.

### 2.5. Cardiac valve replacement

CVR was performed in accordance with the American College of Cardiology/American Heart Association guidelines and indications for valve replacement in patients with valvular heart disease (17). The valve prosthesis type selection was at the discretion of the operating surgeon. All procedures were performed under cardiopulmonary bypass with mild systemic hypothermia (30°C–34°C). Myocardial protection was achieved with cold blood cardioplegia.

### 2.6. Postoperative treatment

Patients were admitted to the ICU as soon as possible following CVR surgery. All patients received standard care, including vital sign monitoring, oxygen therapy, mechanical ventilation, and administration of vasoactive agents. All tests or therapies were considered clinically indicated by the surgical team.

### 2.7. Postoperative outcome assessment

Lengths of stay (hospital, preoperative, postoperative, and ICU), cardiac complications (postoperative arrhythmias, pacemaker use, first dose of dopamine in the ICU, and first dose of dobutamine in the ICU), and respiratory complications (reintubation, pneumonia, and duration of mechanical ventilation) were assessed and compared between the CPAP and non-CPAP treatment groups.

### 2.8. Statistical analysis

The differences between the CPAP and non-CPAP treatment groups of continuous variables were non-normally distributed and tested using the two independent samples Mann–Whitney U test and presented as medians and interquartile ranges. The pre-and postoperative differences for each group were tested by the paired samples Mann–Whitney U test and presented as medians and interquartile ranges. Categorical variables were compared using the chi-square test with a normal approximation or Fisher’s exact test, as appropriate. Two-tailed *p* values <0.05 were considered statistically significant. Statistical analyses were performed with SPSS 22.0 statistical software (IBM^®^ SPSS, Inc., Chicago, IL, United States).

## 3. Results

### 3.1. Baseline participant characteristics

We screened 232 patients with valvular heart disease, 34 of whom with OSA were enrolled and randomized into the CPAP and non-CPAP treatment groups. Fifteen patients in the CPAP treatment group and 17 patients in the non-CPAP treatment group completed the preoperative treatment and postoperative PSG testing (Figure 1). Table 1 shows a comparison of the baseline characteristics between the CPAP and non-CPAP treatment groups. There was no significant difference with respect to age, BMI, sex, atrial fibrillation, heart valve lesions, NYHA class, medication use, LVEF, 6MWT distance, and arterial blood gas results.

**Table 1:** Comparisons of patients’ characteristics.

	CPAP treatment group (*n* = 15)	Non-CPAP treatment group (*n* = 17)	*p*-value
Age (yr)	56 (45–59)	56 (49–61)	0.526
BMI (kg/m^2^)	28.5 (27.4–30.0)	26.8 (25.2–29.7)	0.261
Gender			0.946
Male, *n* (%)	9 (60)	10 (58.8)	
Female, *n* (%)	6 (40)	7 (41.2)	
Heart valve lesions			
Mitral valve lesions, *n* (%)	13 (86.7)	15 (88.2)	1.000
Aortic valve lesions, *n* (%)	9 (60)	10 (58.8)	0.946
Tricuspid valve lesions, *n* (%)	13 (86.7)	8 (47.1)	0.015
NYHA class			0.513
II, *n* (%)	1 (6.7)	3 (17.6)	
III, *n* (%)	10 (66.7)	10 (58.8)	
IV, *n* (%)	4 (26.7)	4 (23.5)	
Atrial fibrillation, *n* (%)	12 (80)	12 (70.6)	0.691
Hypertension	4	6	0.712
Medication use			
Digoxin	12 (80)	13 (76.5)	1.000
Diuretics	13 (86.7)	13 (76.5)	0.659
ACEI/ARB	10 (66.7)	9 (52.9)	0.668
*β*-blockers	8 (53.3)	13 (76.5)	0.266
LVEF (%)	63.4 (58–65.4)	62.5 (58.7–66.2)	0.737
6MWT distance (m)	426 (369–447)	414 (364–460)	0.823
Arterial blood gas			
pH	7.42 (7.36–7.44)	7.43 (7.38–7.46)	0.502
PaO_2_, mmHg	80 (75–86)	81 (72–88)	0.823
PaCO_2_, mmHg	47 (42–48)	44 (41–48)	0.411
ESS score	14 (7–19)	13 (8–16)	0.737

Data are presented as median (interquartile range) or *n* (%). BMI, body mass index, NYHA, New York Heart Association, ACEI, angiotensin-converting enzyme inhibitors, ARB, angiotensin receptor blocker; LVEF, left ventricular ejection fraction; 6MWT, six-minute walking test; ESS, Epworth Sleepiness Scale.

### 3.2. Comparisons of sleep data

The average length of treatment per night is 324 ± 46 min, and the patients had a good adherence with 88% completing >4 h of CPAP treatment per night. The total recording time, total sleep time, sleep efficiency, arousal index, sleep stage, AHI, AI, HI, mean SpO_2_, minimal SpO_2_, and oxygen desaturation index (ODI) were recorded or calculated (Table 2). There was no significant difference in the baseline sleep parameters between the CPAP and non-CPAP treatment groups preoperatively. The AHI, AI, and ODI were significantly decreased, and the mean and minimal SpO_2_ were significantly increased during CPAP treatment. The AHI, AI, and ODI were significantly decreased postoperatively compared with preoperatively (baseline) in the CPAP and non-CPAP treatment groups. There was no significant difference in the sleep parameters between the CPAP and non-CPAP treatment groups postoperatively.

**Table 2:** Comparisons of polysomnography data.

	CPAP treatment group (*n* = 15)				Non-CPAP treatment group (*n* = 17)			*p*-value‡
	Baseline (Preoperative)	CPAP treatment	Postoperative	*p*-value†	Baseline (Preoperative)	Postoperative	*p*-value†	
Total recording time (h)	546 (518–574)	NA	553 (538–573)	0.513	537 (520–560)	551 (522–576)	0.236	0.655
Total sleep time (h)	388 (366–423)	NA	383 (369–403)	0.074	405 (385–414)	387 (364–409)	0.035	0.455
Sleep efficiency (%)	73 (68–76)	NA	68 (67–75)	0.088	74 (70–78)	71 (67–75)	0.076	0.350
Arousal index, h^−1^	21 (18–29)	NA	17 (13–26)	0.005	19 (15–25)	15 (11–22)	0.003	0.655
Sleep stage								
N 1 sleep (%)	14 (12–19)	NA	14 (12–16)	0.105	14 (12–17)	15 (11–16)	0.527	0.551
N 2 sleep (%)	60 (56–63)	NA	61 (58–65)	0.022	58 (56–61)	63 (59–67)	0.004	0.295
N 3 sleep (%)	8 (6–10)	NA	9 (8–12)	0.030	10 (8–12)	7 (5–10)	0.055	0.216
REM sleep (%)	18 (14–21)	NA	17 (12–18)	0.237	16 (13–20)	13 (11–17)	0.046	0.370
AHI, h^−1^	25 (19–33)	3 (1–5)*	19 (14–28)	0017	23 (17–28)	18 (13–27)	0.026	0.766
O-AHI, h^−1^	22 (17–31)	1 (0–2)*	17 (13–25)	0.093	20 (15–25)	18 (11–24)	0.098	0.911
C-AHI, h^−1^	3 (2–5)	2 (1–3)	2 (2–3)	0.073	2 (1–3)	1 (1–3)	0.091	0.132
AI, h^−1^	22 (13–25)	0 (0–1)*	13 (8–18)	0.004	19 (14–22)	14 (10–20)	0.007	0.551
HI, h^−1^	4 (3–11)	3 (1–3)*	6 (5–10)	0.101	4 (3–6)	5 (3–7)	0.408	0.064
Mean SpO_2_ (%)	92 (89–92)	95 (95–97)*	91 (90–92)	0.406	92 (90–94)	92 (91–94)	0.547	0.189
Minimal SpO_2_ (%)	74 (70–79)	88 (84–90)*	77 (73–82)	0.104	72 (67–77)	74 (70–80)	0.016	0.350
ODI, h^−1^	21 (13–27)	2 (1–4)*	15 (12–25)	0.030	19 (13–23)	17 (9–21)	0.019	0.502

Data are presented as median (interquartile range) or *n* (%).

REM, rapid eye movement; AHI, apnea/hypopnea index; AI, apnea index; HI, hypopnea index; SpO_2_, pulse oxygen saturation; ODI, oxygen desaturation index.

* *p* < 0.05, between baseline and CPAP treatment; † *p*-values for within group comparisons, between baseline and postoperative; ‡ *p*-value for postoperative comparisons between groups.

### 3.3. Postoperative complications

Table 3 shows the lengths of postoperative and ICU stays, and the duration of mechanical ventilation were significantly reduced in the CPAP treatment group compared to the non-CPAP treatment group. There was no significant difference in the length of hospital stay, cardiac complications (postoperative arrhythmias, pacemaker use, first dose of dopamine in the ICU, and first dose of dobutamine in the ICU), and respiratory complications (reintubation and pneumonia).

**Table 3:** Comparisons of postoperative complications.

	CPAP treatment group (*n* = 15)	Non-CPAP treatment group (*n* = 17)	*p*-value
Length of stay			
Length of total hospital stay (d)	25 (22–30)	28 (25–36)	0.082
Length of preoperative stay (d)	12 (10–17)	13 (12–17)	0.455
Length of postoperative stay (d)	12 (10–14)	15 (12–18)	**0.044**
Length of ICU stay (h)	22 (17–55)	36 (29–52)	**0.040**
Cardiac complications			
Postoperative arrhythmia, *n* (%)	9 (60)	10 (58.8)	1.000
Pacemaker use, *n* (%)	2 (13.3)	2 (11.8)	1.000
First dose of dopamine in ICU (μg/kg·min)	4 (2–5.5)	4 (3–5)	0.682
First dose of dobutamine in ICU (μg/kg·min)	3 (2–5)	3 (2–4.5)	0.455
Respiratory complications			
Reintubation	1 (6.7)	2 (11.8)	1.000
Pneumonia	2 (13.3)	3 (17.6)	1.000
Duration of mechanical ventilation (h)	17 (12–19)	24 (20–34)	**0.010**

Data are presented as median (interquartile range) or *n* (%).

ICU intensive care unit.

## 4. Discussion

The present study was a novel, randomized controlled trial to determine the effectiveness of preoperative CPAP in patients with OSA undergoing CVR surgery. OSA is a well-documented risk factor associated with perioperative adverse outcomes. Due to recurrent sleep apnea, intermittent hypoxia, and arousal, postoperative adverse events, such as arrhythmias, myocardial injury, and pulmonary infection, may occur. In the Postoperative Vascular Complications in Unrecognized OSA (POSA) study, Chan et al. (6) observed 1,218 patients and concluded that severe OSA was significantly associated with a higher rate of postoperative cardiovascular events. Our previous study showed that patients with OSA were at increased perioperative risk for CVR surgery and associated with overall postoperative recovery, including respiratory insufficiency and a higher rate of postoperative pacemaker use (7).

CPAP treatment during sleep for OSA patients has been shown to significantly reduce AHI and sleepiness, and improve oxygen deficiency (10). Although treatment of OSA appears to reduce postoperative pulmonary complications, pneumonia, and reintubation rates in patients undergoing major abdominal (9) or heart surgery (18, 19), no definitive data have shown whether preoperative CPAP reduces postoperative complications in patients undergoing CVR surgery.

Rennotte et al. (18) reported the effectiveness of CPAP in a case series involving 16 OSA patients. He showed that two OSA patients without preoperative CPAP treatment experienced postoperative adverse events and one patient died, whereas 14 patients with preoperative CPAP treatment had an uneventful postoperative course. Liao et al. (10) developed a randomized controlled trial and confirmed that perioperative auto-CPAP treatment significantly reduced postoperative AHI and improved oxygen saturation in OSA patients. In the present study, the patients in the CPAP group received preoperative auto-CPAP treatment for 1 week. The AHI was decreased and the minimal SpO_2_ was increased significantly during CPAP treatment compared with the preoperative values (25 vs. 3 and 74% vs. 88%; both *p* values <0.05), thus confirming CPAP treatment was effective in reducing sleep apnea and alleviating nocturnal hypoxemia.

A number of studies have shown that postoperative CPAP use significantly reduced the AHI, improves oxygen saturation, and the incidence of postoperative cardiac and pulmonary complications (10–12, 16, 18–23). Due to edema in the upper airway, possible fluid shifts, sedation, and changes in sleep position, postoperative patients, especially patients with OSA, are more likely to have airway obstruction. Theoretically, postoperative CPAP use mitigates these issues as needed. Considering the possible lower adherence to CPAP following CVR surgery, we did not evaluate CPAP postoperatively in both the CPAP and control groups. Even though the postoperative AHI in both groups was slightly reduced (25 vs. 19 and 23 vs. 18; both *p* values <0.05), the reduced AHI may be associated with surgery rather than CPAP treatment because central sleep apnea can be eliminated after CVR (14).

In the present study patients with preoperative auto-CPAP treatment had a shorter duration of postoperative mechanical ventilation, which may further lead to a reduction in ICU and postoperative hospital stays, thus suggesting that reducing the nocturnal hypoxemia and oxygen debt in OSA patients with preoperative CPAP therapy favored a faster postoperative recovery after CVR surgery. A review reported that CPAP treatment reduced the rate of reintubation and admission into the ICU for invasive ventilation and supportive care (24). A matched cohort study showed that OSA patients who received CPAP therapy had a reduction in postoperative cardiovascular complications (25). Kindgen-Milles et al. (26) found that postoperative use of nasal CPAP significantly reduced pulmonary morbidity and the length of hospital stay following surgical repair of thoracoabdominal aortic aneurysms. Our results confirmed these findings in our patients undergoing CVR. CPAP improved upper airway obstruction and increased lung volume, thereby significantly reducing apnea and hypopnea, and the associated hypoxemic and hypercapnic events (11). In addition, the patients treated with CPAP preoperatively may be more familiar and compliant with postoperative mechanical ventilation, which in turn may lead to mechanical ventilation of short duration. Therefore, perioperative CPAP use may be beneficial in patients with OSA who undergo CVR surgery.

The preoperative CPAP treatment did not reduce the incidence of postoperative arrhythmias, pacemaker use, reintubation, and pneumonia in the current study. Zarbock et al. (19) reported that nasal CPAP following cardiac surgery improved arterial oxygenation, reduced the incidence of pulmonary complications, including the pneumonia and reintubation rates, and reduced the readmission rate to the ICU; however, other studies have reported contradictory findings. Altmay et al. (27) observed patients with normal preoperative pulmonary function and CPAP use did not improve lung function after cardiac surgery. Furthermore, short-term CPAP treatment cannot reduce postoperative cardiac arrhythmias (28, 29). There are many factors that induce postoperative arrhythmias, pacemaker use, reintubation, and pneumonia, such as poor cardiac function, hypoxia, and malnutrition. CPAP treatment did not improve all of the aforementioned risk factors. In addition, in our study the small sample size and short time of CPAP treatment were reasons why our data did not reach statistical significance.

CPAP adherence in postoperative patients is low. Liao et al. (11) found that the perioperative adherence rate of CPAP was only 45% and another study (30) found only 33% of the patients had >4 h CPAP treatment; the median adherence was 2.5 h per night. The author speculated that postoperative nausea and vomiting may be associated with reduced CPAP adherence; however, another study did not identify a relationship between the use of CPAP and the increased risk of postoperative nausea and vomiting (31). With better control of postoperative pain and nausea/vomiting, adherence with CPAP among OSA patients may improve (10). Our patients had a good adherence with 88% completing >4 h of CPAP treatment per night. This may be because our patients received short (1 week), preoperative but not postoperative, and in-hospital but not at home CPAP treatment.

## 5. Limitations

There were limitations to our study. First, this study had a short-term clinical observation period, with a 1-week preoperative treatment and 12–15 d postoperative in-hospital outcomes. Although all patients had scheduled follow-up evaluations, we anticipate collecting long-time outcomes in future. Second, even though this was a prospective controlled trial, we did not find significant differences in postoperative arrhythmias, pacemaker use, reintubation, and pneumonia, which may be due to the lack of complications in our study group as a result of the small sample size. Further multicenter and large sample studies are needed. Third, we only observed the effect of preoperative CPAP on postoperative outcomes. Postoperative CPAP use may have a more important role in reduction of the incidence of perioperative cardiac and pulmonary complications. Corollary studies are needed to answer the following important questions: What is the optimal duration of CPAP therapy in OSA patients undergoing CVR surgery? Is CPAP therapy effective preoperatively, postoperatively, or both?

## 6. Conclusion

Preoperative use of auto-CPAP for OSA significantly decreased the duration of mechanical ventilation, and postoperative ICU and hospital stays but failed to show any association with postoperative arrhythmias, pacemaker use, reintubation, and pneumonia.

## Data availability statement

The original contributions presented in the study are included in the article/supplementary material, further inquiries can be directed to the corresponding author.

## Ethics statement

The studies involving human participants were reviewed and approved by Clinical Study Ethics Committee of the First Affiliated Hospital of Nanjing Medical University, No. 2017-SR-040. The patients/participants provided their written informed consent to participate in this study.

## Author contributions

MS and WL collected and analyzed the data and drafted the manuscript. QX and BN enrolled the patients and collected the data. XZ and SZ contributed to the critical revision of the manuscript for important intellectual content. ND designed and managed the study and takes responsibility for the integrity of the data. All authors contributed to the design of the study and the development and implementation of the protocol, critically reviewed the manuscript, approved the final version of the manuscript, and agreed to be responsible for the accuracy and integrity of the work.

## Funding

This work was supported by the National Natural Science Foundation of China (Grant No. 82070093).

## Conflict of interest

The authors declare that the research was conducted in the absence of any commercial or financial relationships that could be construed as a potential conflict of interest.

## Publisher’s note

All claims expressed in this article are solely those of the authors and do not necessarily represent those of their affiliated organizations, or those of the publisher, the editors and the reviewers. Any product that may be evaluated in this article, or claim that may be made by its manufacturer, is not guaranteed or endorsed by the publisher.

## References

[1] Heinzer R Vat S Marques-Vidal P Marti-Soler H Andries D Tobback N. Prevalence of sleep-disordered breathing in the general population: the Hypno Laus study. Lancet Respir Med. (2015) 3:310–8. doi: 10.1016/S2213-2600(15)00043-0, PMID: 25682233 PMC4404207

[2] Punjabi NM. The epidemiology of adult obstructive sleep apnea. Proc Am Thorac Soc. (2008) 5:136–43. doi: 10.1513/pats.200709-155MG, PMID: 18250205 PMC2645248

[3] Pedrosa RP Drager LF Gonzaga CC Sousa MG de Paula LK Amaro AC. Obstructive sleep apnea: the most common secondary cause of hypertension associated with resistant hypertension. Hypertension. (2011) 58:811–7. doi: 10.1161/HYPERTENSIONAHA.111.179788 21968750

[4] Wong JK Maxwell BG Kushida CA Sainani KL Lobato RL Woo YJ. Obstructive sleep apnea is an independent predictor of postoperative atrial fibrillation in cardiac surgery. J Cardiothorac Vasc Anesth. (2015) 29:1140–7. doi: 10.1053/j.jvca.2015.03.024, PMID: 26154572

[5] Gami AS Olson EJ Shen WK Wright RS Ballman KV Hodge DO. Obstructive sleep apnea and the risk of sudden cardiac death: a longitudinal study of 10,701 adults. J Am Coll Cardiol. (2013) 62:610–6. doi: 10.1016/j.jacc.2013.04.080, PMID: 23770166 PMC3851022

[6] Chan MTV Wang CY Seet E Tam S Lai HY Chew EFF. Association of Unrecognized Obstructive Sleep Apnea with Postoperative Cardiovascular Events in patients undergoing major noncardiac surgery. JAMA. (2019) 321:1788–98. doi: 10.1001/jama.2019.4783, PMID: 31087023 PMC6518343

[7] Ding N Ni BQ Wang H Ding WX Xue R Lin W. Obstructive sleep apnea increases the perioperative risk of cardiac valve replacement surgery: a prospective single-center study. J Clin Sleep Med. (2016) 12:1331–7. doi: 10.5664/jcsm.6182, PMID: 27448416 PMC5033734

[8] Abdelsattar ZM Hendren S Wong SL Campbell DA Jr Ramachandran SK. The impact of untreated obstructive sleep apnea on cardiopulmonary complications in general and vascular surgery: a cohort study. Sleep. (2015) 38:1205–10. doi: 10.5665/sleep.4892, PMID: 25761980 PMC4507725

[9] Ferreyra GP Baussano I Squadrone V Richiardi L Marchiaro G Del Sorbo L. Continuous positive airway pressure for treatment of respiratory complications after abdominal surgery: a systematic review and meta-analysis. Ann Surg. (2008) 247:617–26. doi: 10.1097/SLA.0b013e3181675829 18362624

[10] Liao P Luo Q Elsaid H Kang W Shapiro CM Chung F. Perioperative auto-titrated continuous positive airway pressure treatment in surgical patients with obstructive sleep apnea: a randomized controlled trial. Anesthesiology. (2013) 119:837–47. doi: 10.1097/ALN.0b013e318297d89a, PMID: 24195872

[11] Nagappa M Mokhlesi B Wong J Wong DT Kaw R Chung F. The effects of continuous positive airway pressure on postoperative outcomes in obstructive sleep apnea patients undergoing surgery: a systematic review and Meta-analysis. Anesth Analg. (2015) 120:1013–23. doi: 10.1213/ANE.0000000000000634 25899270

[12] Kong WT Chopra S Kopf M Morales C Khan S Zuccala K. Perioperative risks of untreated obstructive sleep apnea in the bariatric surgery patient: a retrospective study. Obes Surg. (2016) 26:2886–90. doi: 10.1007/s11695-016-2203-3, PMID: 27206775

[13] Remenyi B Wilson N Steer A Ferreira B Kado J Kumar K. World heart federation criteria for echocardiographic diagnosis of rheumatic heart disease--an evidence-based guideline. Nat Rev Cardiol. (2012) 9:297–309. doi: 10.1038/nrcardio.2012.7, PMID: 22371105 PMC5523449

[14] Ding N Ni BQ Zhang XL Zha WJ Hutchinson SZ Lin W. Elimination of central sleep apnea by cardiac valve replacement: a continuous follow-up study in patients with rheumatic valvular heart disease. Sleep Med. (2014) 15:880–6. doi: 10.1016/j.sleep.2014.02.007, PMID: 24938583

[15] Berry RB Budhiraja R Gottlieb DJ Gozal D Iber C Kapur VK. Rules for scoring respiratory events in sleep: update of the 2007 AASM manual for the scoring of sleep and associated events. Deliberations of the sleep apnea definitions task force of the American Academy of sleep medicine. J Clin Sleep Med. (2012) 08:597–619. doi: 10.5664/jcsm.2172, PMID: 23066376 PMC3459210

[16] O'Gorman SM Gay PC Morgenthaler TI. Does autotitrating positive airway pressure therapy improve postoperative outcome in patients at risk for obstructive sleep apnea syndrome? A randomized controlled clinical trial. Chest. (2013) 144:72–8. doi: 10.1378/chest.12-0989, PMID: 23287823

[17] American College of C, American Heart Association Task Force on Practice G, Society of Cardiovascular A Bonow RO Carabello BA Chatterjee K de Leon AC Faxon DP. ACC/AHA 2006 guidelines for the management of patients with valvular heart disease: a report of the American College of Cardiology/American Heart Association task force on practice guidelines (writing committee to revise the 1998 guidelines for the management of patients with valvular heart disease) developed in collaboration with the Society of Cardiovascular Anesthesiologists endorsed by the Society for Cardiovascular Angiography and Interventions and the Society of Thoracic Surgeons. J Am Coll Cardiol. (2006) 48:e1–e148. doi: 10.1016/j.jacc.2006.05.021 16875962

[18] Rennotte MT Baele P Aubert G Rodenstein DO. Nasal continuous positive airway pressure in the perioperative management of patients with obstructive sleep apnea submitted to surgery. Chest. (1995) 107:367–74. doi: 10.1378/chest.107.2.367, PMID: 7842763

[19] Zarbock A Mueller E Netzer S Gabriel A Feindt P Kindgen-Milles D. Prophylactic nasal continuous positive airway pressure following cardiac surgery protects from postoperative pulmonary complications: a prospective, randomized, controlled trial in 500 patients. Chest. (2009) 135:1252–9. doi: 10.1378/chest.08-1602, PMID: 19017864

[20] Gupta RM Parvizi J Hanssen AD Gay PC. Postoperative complications in patients with obstructive sleep apnea syndrome undergoing hip or knee replacement: a case-control study. Mayo Clin Proc. (2001) 76:897–905. doi: 10.4065/76.9.897 11560300

[21] Jensen C Tejirian T Lewis C Yadegar J Dutson E Mehran A. Postoperative CPAP and BiPAP use can be safely omitted after laparoscopic roux-en-Y gastric bypass. Surg Obes Relat Dis. (2008) 4:512–4. doi: 10.1016/j.soard.2008.05.003, PMID: 18656832

[22] Liao P Yegneswaran B Vairavanathan S Zilberman P Chung F. Postoperative complications in patients with obstructive sleep apnea: a retrospective matched cohort study. Can J Anaesth. (2009) 56:819–28. doi: 10.1007/s12630-009-9190-y 19774431

[23] Goucham AB Coblijn UK Hart-Sweet HB de Vries N Lagarde SM van Wagensveld BA. Routine postoperative monitoring after bariatric surgery in morbidly obese patients with severe obstructive sleep apnea: ICU admission is not necessary. Obes Surg. (2016) 26:737–42. doi: 10.1007/s11695-015-1807-3, PMID: 26210193

[24] Ireland CJ Chapman TM Mathew SF Herbison GP Zacharias M. Continuous positive airway pressure (CPAP) during the postoperative period for prevention of postoperative morbidity and mortality following major abdominal surgery. Cochrane Database Syst Rev. (2014) 2014:CD008930. doi: 10.1002/14651858.CD008930.pub2, PMID: 25081420 PMC6464713

[25] Mutter TC Chateau D Moffatt M Ramsey C Roos LL Kryger M. A matched cohort study of postoperative outcomes in obstructive sleep apnea: could preoperative diagnosis and treatment prevent complications? Anesthesiology. (2014) 121:707–18. doi: 10.1097/ALN.0000000000000407 25247853

[26] Kindgen-Milles D Muller E Buhl R Bohner H Ritter D Sandmann W. Nasal-continuous positive airway pressure reduces pulmonary morbidity and length of hospital stay following thoracoabdominal aortic surgery. Chest. (2005) 128:821–8. doi: 10.1378/chest.128.2.821, PMID: 16100174

[27] Altmay E Karaca P Yurtseven N Ozkul V Aksoy T Ozler A. Continuous positive airway pressure does not improve lung function after cardiac surgery. Can J Anaesth. (2006) 53:919–25. doi: 10.1007/BF03022835 16960270

[28] Campos J Ernst G Blanco M Cassano A Tello-Santa-Cruz I Caceres-Monie C. Acute response to 7-day therapy with CPAP in patients with moderate to severe obstructive sleep apnea and cardiac arrhytmia. Sleep Sci. (2018) 11:49–53. doi: 10.5935/1984-0063.20180011, PMID: 29796202 PMC5916577

[29] Caples SM Mansukhani MP Friedman PA Somers VK. The impact of continuous positive airway pressure treatment on the recurrence of atrial fibrillation post cardioversion: a randomized controlled trial. Int J Cardiol. (2019) 278:133–6. doi: 10.1016/j.ijcard.2018.11.100, PMID: 30522886

[30] Guralnick AS Pant M Minhaj M Sweitzer BJ Mokhlesi B. CPAP adherence in patients with newly diagnosed obstructive sleep apnea prior to elective surgery. J Clin Sleep Med. (2012) 08:501–6. doi: 10.5664/jcsm.2140, PMID: 23066360 PMC3459194

[31] Meng L. Postoperative nausea and vomiting with application of postoperative continuous positive airway pressure after laparoscopic gastric bypass. Obes Surg. (2010) 20:876–80. doi: 10.1007/s11695-008-9741-2, PMID: 18925384

